# A Sensitive Gold Nanoplasmonic SERS Quantitative Analysis Method for Sulfate in Serum Using Fullerene as Catalyst

**DOI:** 10.3390/nano8050277

**Published:** 2018-04-26

**Authors:** Chongning Li, Libing Wang, Yanghe Luo, Aihui Liang, Guiqing Wen, Zhiliang Jiang

**Affiliations:** 1 Key Laboratory of Ecology of Rare and Endangered Species and Environmental Protection, (Guangxi Normal University), Ministry of Education, Guangxi Key Laboratory of Environmental Pollution Control Theory and Technology, Guilin 541004, China; lcn7882342@163.com (C.L.); 18074841309@163.com (L.W.); ahliang2008@163.com (A.L.); 2 School of Food and Bioengineering, Hezhou University, Hezhou 542899, China; kira0217@foxmail.com

**Keywords:** sulfate, fullerene catalysis, gold nanoplasmon, surface-enhanced Raman scattering (SERS)

## Abstract

Fullerene exhibited strong catalysis of the redox reaction between HAuCl_4_ and trisodium citrate to form gold nanoplasmon with a strong surface-enhanced Raman scattering (SERS) effect at 1615 cm^−1^ in the presence of Vitoria blue B molecule probes. When fullerene increased, the SERS peak enhanced linearly due to formation of more AuNPs as substrate. Upon addition of Ba^2+^, Ba^2+^ ions adsorb on the fullerene surface to inhibit the catalysis of fullerene that caused the SERS peak decreasing. Analyte SO_4_^2−^ combined with Ba^2+^ to form stable BaSO_4_ precipitate to release free fullerene that the catalysis recovered, and the SERS intensity increased linearly. Thus, a new SERS quantitative analysis method was established for the detection of sulfate in serum samples, with a linear range of 0.03–3.4 μM.

## 1. Introduction

Surface plasmon polaritons (SP) are the collective oscillatory behavior of free electrons on metal surfaces when light wave incidentd on the interface between the metal and medium. When surface plasmon is localized on the surface of metal nanoparticles with particle sizes far less than the wavelength of incident light, localized surface plasmon resonance (SPR) is the resonant oscillation of conduction electrons at the nanosurface that are excited by the light [1]. Combined with its good biocompatibility and the mature functional modification of biological molecules surface, the SPR effect has given excellent physical and chemical properties to noble metal nanomaterials such as gold, silver and copper. Recently, it has become a promising technology for nanosensing, bioimaging, analytical separation and biomedical research [1,2]. Among various nanomaterials, gold nanoparticles are widely used to construct visual sensors due to their unique SPR optical properties [2]. The nanoparticle resonance scattering effect is one of the important applications of nanoplasmon in analytical chemistry. It includes elastic resonance Rayleigh scattering (RRS) and inelastic surface-enhanced Raman scattering (SERS). RRS spectroscopy is simple, easy and sensitive, and has been used for the analysis of trace proteins, nucleic acids and heavy metals [3]. Short-chain DNA has been generated from DNA enzymes by lead ion-catalytic cleavage, which protects the gold nanoparticles from aggregation by NaCl. Non-aggregated gold nanoparticles also have a catalytic effect on HAuCl_4_-H_2_O_2_ reaction, and trace amounts of lead ion can be detected by RRS. However, a high concentration of NaCl was used as aggregating agent, and large-size gold nanoparticles such as gold nanoflower were not suitable for the system [4]. SERS is one of the most direct applications of nanoplasmon [5,6,7]. This powerful molecular spectral technique is based on the enhancement of inelastic scattering of plasma-excited and surface-adsorbed molecules upon irradiation of the nanostructured surface plasmons. It is one of the few available molecular detection techniques [7,8]. SERS detection probes can be constructed by modifying Raman reporter molecules and target capture molecules on the surface of noble metal nanoparticles. The high specificity and high sensitivity of DNA, proteins and other molecules can be detected by the specific effect of target capture molecules [9]. The formation of further enhanced localized “hot spots” by interparticle coupling further enhances the Raman enhancement factor. Wang et al. [10] used double-modified gold nanoparticles and an antigen–antibody mediated self-assembly sandwich structure formed by interaction between particles to form a “hot spot” to achieve high-specificity multiplex detection of three cytokines in complex biological systems. In recent years, SERS quantitative analysis has greatly improved [11,12,13,14,15], especially in the preparation of sensitive and reproducible nanosol substrates. Liang et al. [11] used H_2_O_2_, NaBH_4_ and citric acid as a reducing agent to prepare silver nanorods/reduced graphene oxide (AgNR/rGO) nanosol substrate with good stability to detect 8–1500 nM iodide ion. Yang et al. [12] reported silver nanoparticles as a substrate to determine SCN^−1^ in milk powder by the SERS method, with a linear range of 2–191.0 mg/L SCN^−1^. Luo et al. [13] prepared triangular nanosilver using graphene oxide as catalyst, and SERS quantitative analysis of 0.7–72 nM nitrite. Zhang et al. [14] reported a SERS method for sulfur dioxide in food, with the lowest detectable concentration of 1 mg kg^−1^, based on the S atom Raman peak at 630 cm^−1^. Shang et al. [15] used AgNO_3_ as the precursor to prepare stable silver nanochain (AgNC) sol as a SERS substrate and analyzed 0.0125–0.3 μM sodium hexametaphosphate. However, for anions such as sulfate, whose Raman-scattering section is small, the sensitivity of direct detection is too low to be detected. To the best of our knowledge, there are no SERS quantitative analysis methods for trace sulfates. Therefore, it is of great importance to develop a new SERS quantitative assay for small anions such as sulfate based on the fullerene catalytic generation of nanoplasmon.

Fullerene (C_60_) is a very important carbon nanomaterial that is applied in the field of solar energy-conversion materials, catalysis and analytical science [16,17,18]. C_60_ is a hydrophobic carbon nano-material, its ability to dissolve in water is very low, and it easily accumulates in water, which limits its application. For this reason, researchers usually modify the surface of C_60_ to enhance its water solubility so as to obtain better applications. Lanzellotto et al. [19] used fullerene as a bridge, through the fullerene surface alcohol-connected electrode-AuNP and laccase (TvL) to construct an electrochemical biosensor for detection of 0.03–0.30 mM tea polyphenols in beer. Lu et al. [20] inserted fullerol into mercaptoporphyrins and monolayer polyaniline films to prepare electrochemical molecular probes to detect 0.029–10,000 nM benzene. Hang et al. [21] covalently bonded fullerol-rich hydroxyls to thioglycolic acid and PO_4_^3−^ of DNA to construct a DNA molecular probe to detect 1–1000 fM DNA. Wu et al. [22] constructed a zinc porphyrin–fullerene derivative based non-enzymatic electrochemical sensor for sensing of 0.035 to 3.40 mM H_2_O_2_. Li et al. [23] reported a colorimetric sensor based on the intrinsic peroxidase-like activity of C_60_-carboxy fullerenes toward 1.0–40 μM glucose, that catalyzed the colored reaction of H_2_O_2_ and 3,3′,5,5′-tetramethyl benzidine (TMB). Bhim et al. [24] synthesized a water-compatible fullerene-monoadduct to determine 1.47–247.2 ng mL^−1^ chlorambucil electrochemically. To date, there have been no reports about the use of SERS in the determination of trace SO_4_^2−^ based on the BaSO_4_ reaction mediating C_60_ catalytic gold nanoplasmons. 

Sulfate ions play a very important role in life and environmental science. After entering the environment, these will pollute it and cause harm to the human body [25]. Therefore, selective and highly sensitive detection of sulfate ions in biological and environmental samples is of great significance. At present, several methods, including ion chromatography (IC), chemiluminescence, spectrophotometry and atomic absorption spectrometry, have been reported for the determination of sulfate ions [26,27,28,29,30]. Among them, IC is a good method, but its sensitivity is low. Therefore, it is important to develop highly sensitive and selective methods for sulfate ions using the new technology of SERS and the new material of fullerene nanocatalyst. In this article, a new and sensitive SERS quantitative analysis method was developed for the determination of sulfate, coupling the BaSO_4_ reaction and C_60_ catalytic reaction of HAuCl_4_-trisodium citrate.

## 2. Results and Discussion

### 2.1. Analytical Principle

**Figure 1:**
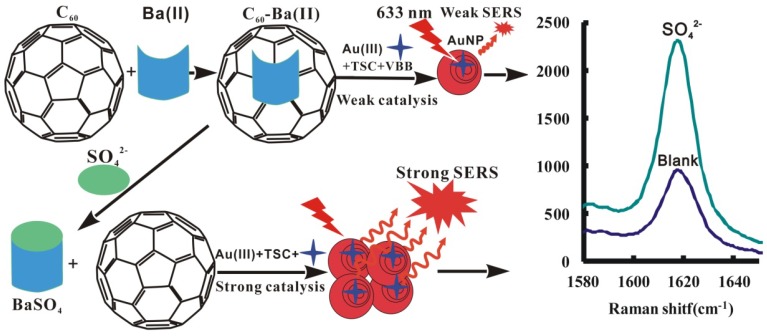
SERS detection of sulfate combined BaSO_4_ reaction with nanogold reaction.

At 60 °C, the AuNP reaction between HAuCl_4_ and trisodium citrate (TSC) is very slow, and C_60_ exhibits strong catalysis of the AuNP reaction. Ba^2+^ ions adsorb on C_60_ surface to inhibit the AuNP reaction. Upon addition of SO_4_^2−^, stable BaSO_4_ precipitates form to escape free and for C_60_ catalysis recovery, causing the SERS peak to increase due to the formation of more nanosol substrate of AuNPs when molecular probes of Victoria blue B (VBB) was added. On these grounds, a new SERS quantitative analysis was established for the detection of trace sulfate (Figure 1).

### 2.2. Surface-Enhanced Raman Scattering (SERS) Spectra

**Figure 2:**
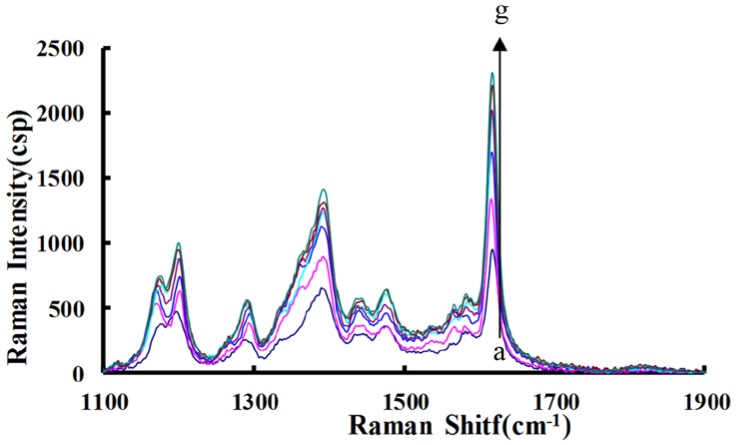
Surface-enhanced Raman scattering (SERS) spectra of HAuCl_4_-TSC-C_60_-Na_2_SO_4_-BaCl_2_-VBB system. a: 4.2 μM HAuCl_4_ + 0.33 μM VBB + 0.33 mg/L C_60_ + 170 μM TSC + 53 μM BaCl_2_; b: a + 0.33 μM Na_2_SO_4_; c: a + 0.67 μM Na_2_SO_4_; d: a + 1 μM Na_2_SO_4_; e: a + 1.33 μM Na_2_SO_4_; f: a + 1.98 μM Na_2_SO_4_; g: a + 2.31 μM Na_2_SO_4_.

C_60_ and C_60_OH analytical systems were examined by the SERS technique using VBB as molecular probes. There are three strong SERS peaks at 1202 cm^−1^, 1394 cm^−1^ and 1615 cm^−1^. When SO_4_^2−^ concentration increased, SERS signals increased greatly due to the formation of more AuNPs. For the two analytical systems (Figure 2), the C_60_ system is the most sensitive and the peak at 1615 cm^−1^ is strongest. Thus, the C_60_ system with a SERS peak at 1615 cm^−1^ was chosen to detect SO_4_^2−^.

### 2.3. Transmission Electron Microscopy (TEM)

**Figure 3:**
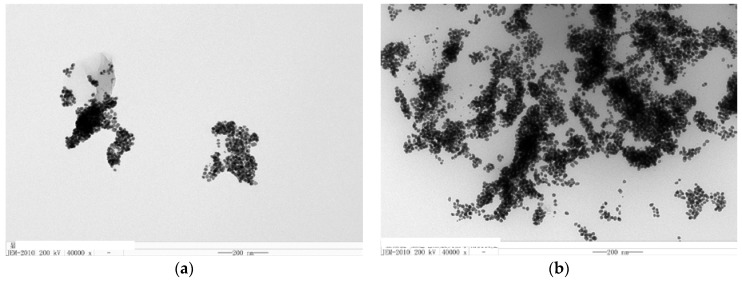
Transmission electron microscopy (TEM) of the analytical system. (**a**) 4.2 μM HAuCl_4_ + 0.33 μM VBB + 0.33 mg/L C_60_ + 170 μM TSC + 53 M BaCl_2_; (**b**) a + 1.67 μM Na_2_SO_4_.

Transmission electron microscopy (TEM) of HAuCl_4_-TSC-C_60_-Na_2_SO_4_-BaCl_2_-VBB analytical system was undertaken. In the absence of Na_2_SO_4_, the AuNP reaction is very slow and formed few spherical AuNPs of an average size of 20 nm (Figure 3). When Na_2_SO_4_ was added, more AuNPs of an average size of 15 nm formed due to recovering C_60_ catalytic activity that caused the SERS signal to be enhanced. 

### 2.4. Optimization of Analytical Conditions

**Figure 4:**
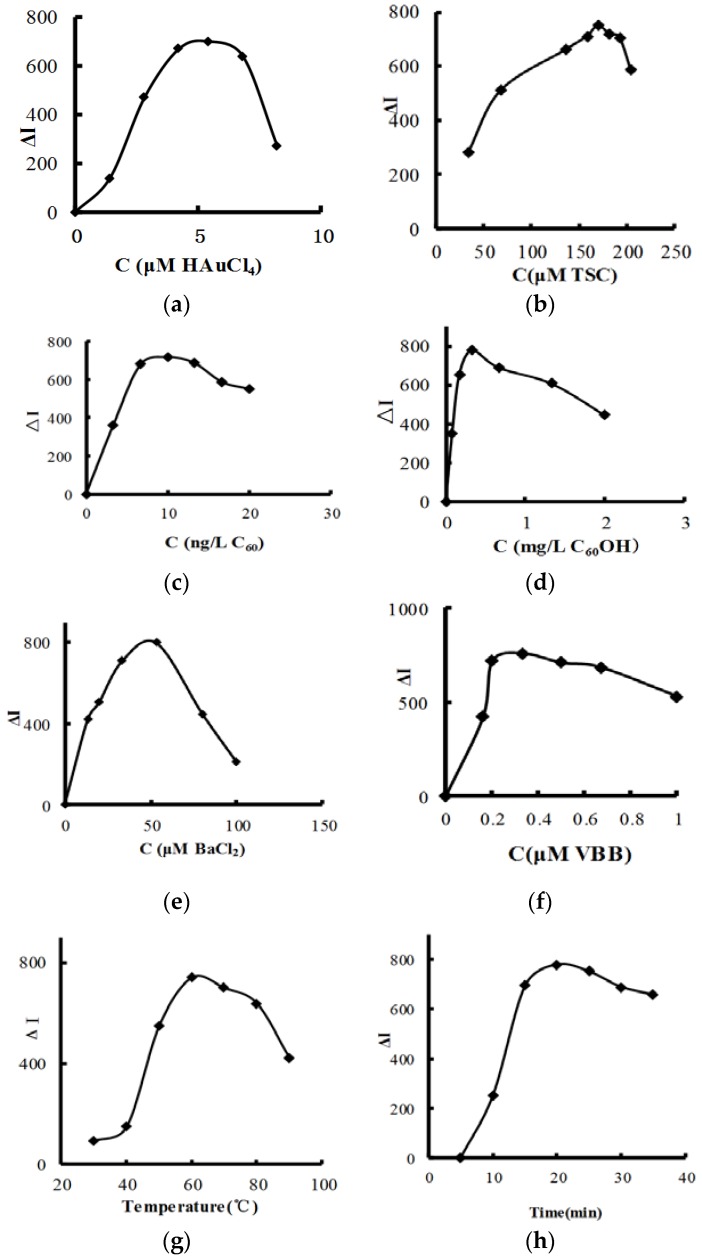
Effect of reagent concentration, reaction temperature and time: (**a**) HAuCl_4_ + 0.33 μM VBB + 0.33 mg/L C_60_ + 170 μM TSC + 0.67 μM Na_2_SO_4_ + 53 μM BaCl_2_; (**b**) TSC + 4.2 μM HAuCl_4_ + 0.33 μM VBB + 0.33 mg/L C_60_ + 0.67 μM Na_2_SO_4_ + 53 μM BaCl_2_; (**c**) C_60-OH_ + 170 μM TSC + 4.2 μM HAuCl_4_ + 53 μM BaCl_2_ + 0.33 μM VBB + 0.67 μM Na_2_SO_4_; (**d**) C_60_ + 170 μM TSC + 4.2 μM HAuCl_4_ + 53 μM BaCl_2_ + 0.33 μM VBB + 0.67 μM Na_2_SO_4_; (**e**) BaCl_2_ + 170 μM TSC + 4.2 μM HAuCl_4_ + 0.33 mg/L C_60_ + 0.33 μM VBB + 0.67 μM Na_2_SO_4_; (**f**) VBB + 0.67 μM Na_2_SO_4_ + 53 μM BaCl_2_ + 170 μM TSC + 4.2 μM HAuCl_4_ + 0.33 mg/L C_60_; (**g**) 0.67 μM Na_2_SO_4_ + 53 μM BaCl_2_ + 170 μM TSC + 4.2 μM HAuCl_4_ + 0.33 mg/L C_60_ + 0.33 μM VBB; (**h**) 0.67 μM Na_2_SO_4_ + 53 μM BaCl_2_ + 170 μM TSC + 4.2 μM HAuCl_4_ + 0.33 mg/L C_60_ + 0.33 μM VBB.

According to the procedure, the following parameters were optimized: concentration of HAuCl_4_, TSC, C_60_, C_60_OH, BaCl_2_ and VBB, reaction temperature and time. Respective data and figures are given in Figure 4. The following conditions were found to give the best results: 4.2 μM HAuCl_4_, 170 μM TSC, 0.33 mg/L C_60_, 9.99 ng/L C_60_OH, 53 μM BaCl_2_ and 0.33 μM VBB, and a reaction temperature of 60 °C for 20 min.

### 2.5. Working Curve

**Figure 5:**
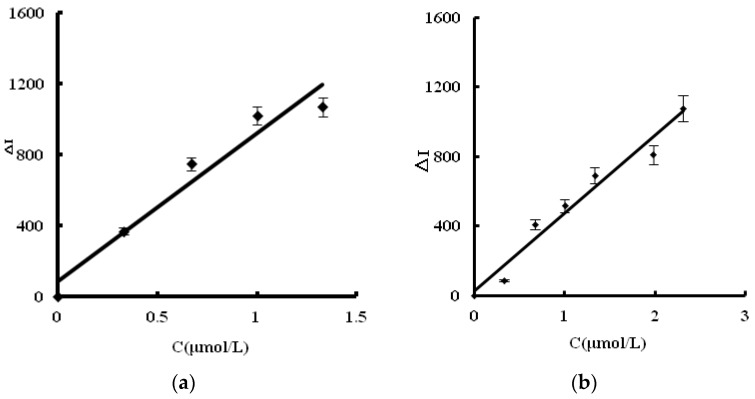
SERS working curve. (**a**) C_60_; (**b**) C_60_OH.

For the C_60_ analytical system, SERS intensity was linear to the SO_4_^2−^ concentration in the 0.03–2.31 μM linear range (LR), with a regression equation of ΔI = 550.3C + 23.3, coefficient of 0.9474 and detection limit (DL) of 0.01 μM (Figure 5a). For the C_60-OH_ system, the SERS intensity was linear to the SO_4_^2−^ concentration in the 0.06–2.31 μM range, with a regression equation of ΔI = 447.9C + 24.7, coefficient of 0.9635, and DL of 0.03 μM (Figure 5b). The C_60_ is more sensitive than the C_60_OH, and was selected for the detection of sulfate. In short, this article was utilized advance nanoplasmonic SERS technique and nanoreaction to develop a highly sensitive SERS quantitative analysis method for sulfate ions that firstly used VBB as label-free molecular probe and C_60_ as nanocatalyst for AuNP reaction between HAuCl_4_ and trisodium citrate. In a comparison of reported methods [26,27,28,29,30] for sulfate determination (Table 1), this new SERS quantitative analysis method for sulfate is one of most sensitive methods, with less serum sample used.

### 2.6. Influence of Interfering Ions

The interference of 16 coexisting substances on the determination of 0.66 µM SO_4_^2−^ was investigated according to the procedure. Results (Table 2) show that common substances did not interfere with the determination. This indicated that the SERS method has good selectivity.

### 2.7. Analysis of Samples

Total sulfur in whole blood included inorganic sulfate ions and various non-inorganic sulfates [30]. This proposed SERS method was applied to the determination of inorganic sulfates in human blood serum samples collected from three healthy people. Trichloroacetic acid was added in a 0.10 mL serum and 9 mL water to remove proteins by centrifugation at 7000 r/min for 10 min, and was diluted to 10 mL to obtain the sample solution. Sulfate content was determined five times to obtain a single value according to the procedure outlined in Section 3.3 below, with relative standard deviation (RSD) of 1.9–4.2%. 0.300 μg/mL sulfate was added in three samples respectively, and determined the sulfate concentration. Then, recovery was calculated and was between 95.0% and 99.3% (Table 3). According to a dilution time of 100 and average values, the content of sulfate in serum was between 36.9 μg/mL and 41.9 μg/mL.

## 3. Materials and Methods

### 3.1. Apparatus

The following were used: DXR model smart Raman spectrometer (Thermo, Waltham, MA, USA) with laser wavelength of 633 nm, power of 3.5 mW, slit of 50 μm and acquisition time of 5 s; 3K-15 high-speed refrigerated centrifuge model (Sigma Co., Darmstadt, Germany); 79-1 magnetic stirrer with heating model (Zhongda Instrumental Plant, Jiangsu, China) HH-S2 electric hot water bath model (Earth Automation Instrument Plant, Jintan, China) BAO-150A precision blast oven model (Shi Dukai Equipment Co., Ltd., Shanghai, China); S-4800 field emission scanning electron microscope (Hitachi High-Technologies Corporation, Japan/Oxford Company, Oxford, UK); and SYZ-550 quartz sub-boiling distilled water model (Crystal Glass Instrument Plant, Jiangsu, China).

### 3.2. Reagents

2.9 mM HAuCl_4_ (National Pharmaceutical Group Chemical Reagents Company, Shanghai, China, http://www.reagent.com.cn); 10 μM VBB (Shanghai Reagent Three Factory, Shanghai, China) stock solution; 1.0 mM BaCl_2_ (Hunan Reagent Factory, Changsha, China); 1.00 mM Na_2_SO_4_ (Xilong Science Co., Ltd., Shantou, China); and 3.4 mM trisodium citrate (Xilong Chemical Plant, Shantou, China) were prepared. Fullerene solution C_60_: A 0.02 g C_60_ was dissolved in 20 mL toluene by ultrasonic waves to obtain a bright purple solution. Then, 100 mL water was added and placed in an ultrasonic instrument to volatilize all toluene and so prepare a deep yellow suspension with concentration of 0.2 g/L C_60_. Fullerol (C_60_OH) solution: an accurately weighed 0.2 g C_60_OH was dissolved in 100 mL water to obtain a concentration of 2 g/L C_60_OH.

### 3.3. Procedure

In a 5 mL test tube, a suitable amount of Na_2_SO_4_, 80 μL 1 mM BaCl_2_ and 25 μL 20 mg/L C_60_ were added and mixed well. Then, 100 μL 0.1% HAuCl_4_ and 75 μL 3.4 mM TSC solution were added and diluted to 1.5 mL. The mixture was heated to 60 °C in a water bath for 20 min, cooled with ice-water, and 50 μL 10 μM VBB was added. The Raman spectrum was recorded by a scanning Raman spectrometer. The SERS intensity at 1615 cm^−1^ (${}_{I_{{1615\;\mathrm{cm}}^{-1}}}$) and blank value (${}_{I_{{1615\;\mathrm{cm}}^{-1}}}$)_0_ without sulfate were measured. The ΔI = ${}_{I_{{1615\;\mathrm{cm}}^{-1}}}$ − (${}_{I_{{1615\;\mathrm{cm}}^{-1}}}$)_0_ value was calculated.

## 4. Conclusions

C_60_ exhibited a strong catalysis of reduction of HAuCl_4_ by trisodium citrate to form high SERS-active AuNPs. Ba(II) ions can combine with C_60_ to produce Ba-C_60_ complexes to inhibit the nanocatalysis. Upon the addition of sulfate ions, stable BaSO_4_ precipitate formed to release C_60_, which activated the catalytic effect of C_60_ and enhanced the SERS peak linearly. Thus, a new SERS quantitative analysis method was established for the determination of trace sulfate in serum samples, with simplicity, high sensitivity and selectivity, and less serum sample consumption.

## Figures and Tables

**Table 1:** Comparison of analytical methods for determination of SO_4_^2+^.

Method	Principle	LR (mg/L)	DL (mg/L)	Comments	Ref.
IC	Determination of sulphate in concentrated nitric acid.	0.7–5	0.5	Complex operation, low sensitivity.	[26]
FIA–CL	Combination of FIA-CL with ion-exchanger for detection of sulphate in water.	48–960	-	Simple, but low sensitivity.	[27]
IC	Detection of sulphate in salt with conductivity detection.	0.05–10	0.05	Sensitivity.	[28]
SFI	SFI determination of sulphate in soil solutions at 668 nm.	0.25–1.5	0.1	Narrow LR, low sensitivity.	[29]
AAS	Determination of inorganic plasma sulfate by indirect AAS.	0.14–1.12	0.003	Sensitive.	[30]
SERS	Combination of Ni(II) complex with CD catalyzing the AuNP reaction with VBB molecular probes.	0.0028–0.163	9.3 × 10^−4^	Simple, rapid, sensitive, selective.	This method

IC: ion chromatography; FIA–CL: flow-injection analysis–cheminluminescence; SFI: spectrophotometric flow-injection; AAS: atomic absorption spectrophotometry.

**Table 2:** Effect of coexistence substances.

Coexistent Substance	Times	Relative Error (%)	Coexistent Substance	Times	Relative Error (%)
Zn^2+^	100	4.0	Mg^2+^	100	8.0
Ca^2+^	100	−1.0	glycol	100	8.0
Pb^2+^	100	6.0	Cr^6+^	30	6.0
NH_4_Cl	100	2.0	Fe^3+^	20	−4.0
Cu^2+^	100	1.2	NO_2_^−^	40	−5.0
K^+^	100	2.0	HSA	100	3.0
Bi^3+^	100	−4.0	Mn^2+^	10	−6.0
SO_3_^2−^	100	7.0	alcohol	100	−5.0

**Table 3:** Results of samples analysis.

Serum	Single Value (μg/mL)	Average (μg/mL)	Added (μg/mL)	Found (μg/mL)	Recovery (%)	RSD (%)	Content (μg/mL)
No 1	0.39, 0.41, 0.38, 0.40, 0.43	0.402	0.300	0.700	99.3	4.2	40.2
No 2	0.410, 0.410, 0.418, 0.424, 0.434	0.419	0.300	0.710	97.0	1.9	41.9
No 3	0.360, 0.368, 0.384, 0.365, 0.369	0.369	0. 300	0.654	95.0	2.4	36.9

## References

[1] Su Y., Peng T., Yin F., Li D., Fan C. (2017). Nanoplasmonic biological sensing and imaging. Acta Chim. Sin..

[2] Ma X., Sun M., Lin Y., Liu Y., Luo F., Guo L., Qiu B., Lin Z., Chen G. (2018). Progress of visual biosensor based on gold nanoparticles. Chin. J. Anal. Chem..

[3] Wen G., Liang A., Jiang Z. (2013). Functional nucleic acid nanoparticle-based resonance scattering spectral probe. Plasmonics.

[4] Wang Y., Wen G., Ye L., Liang A., Jiang Z. (2016). Label-free SERS study of galvanic replacement reaction on silver nanorod surface and its application to detect trace mercury ion. Sci. Rep..

[5] Li C., Fan P., Liang A., Liu Q., Jiang Z. (2018). Aptamer based determination of Pb(II) by SERS and by exploiting the reduction of HAuCl_4_ by H_2_O_2_ as catalyzed by grapheme oxide nanoribbons. Microchim. Acta.

[6] Luo L., Chen Y., Zhang L., Li Y., Li H., Zhang H., Tian Y. (2017). SERS assay for pyrophosphate based on its competitive binding to Cu(II) ion on silver nanoparticles modified with cysteine and rhodamine 6G. Microchim. Acta.

[7] Yang T., Yang H., Zhen S., Huang C. (2015). Hydrogen-bond-mediated in situ fabrication of AgNPs/Agar/PAN electrospun nanofibers as reproducible SERS substrates. ACS Appl. Mater. Interfaces.

[8] Fan M., Cheng F., Wang C., Gong Z., Tang C., Man C., Brolo A.G. (2015). SERS Optrode as a “fishing rod” to direct pre-concentrate analytes from superhydrophobic surfaces. Chem. Commun..

[9] Lee J.H., Nam J.M., Jeon K.S., Lim D.K., Kim H., Kwon S., Lee H., Suh Y.D. (2012). Tuning and maximizing the single-molecule surface-enhanced Raman scattering from DNA-tethered nanodumbbells. ACS Nano.

[10] Wang Y., Tang L., Jiang J. (2013). SERS-based, homogeneous, multiplexed immunoassay with antibody-fragments decorated dold nanoparticles. Anal. Chem..

[11] Liu Q., Zhang X., Wen G., Luo Y., Liang A., Jiang Z. (2015). A sensitive silver nanorod/reduced graphene oxide SERS analytical platform and its application to quantitative analysis of iodide in solution. Plasmonics.

[12] Yang Q., Liang F., Wang D., Ma P., Gao D., Han J., Li Y., Yu A., Song D., Wang X. (2014). Simultaneous determination of thiocyanate ion and melamine in milk and milk powder using surface-enhanced Raman spectroscopy. Anal. Methods.

[13] Luo Y., Wen G., Dong J., Liu Q., Liang A., Jiang Z. (2014). SERS detection of trace nitrite ion in aqueous solution based on the nitrosation reaction of rhodamine 6G molecular probe. Sens. Actuators B.

[14] Zhang L., Zeng Y., Zhao J., Chen H., Kong J., Chen Q., Lin H., Tian Z., Liu G. (2017). Rapid determination of sulfur dioxide residues in foods based on surface-enhanced Raman spectroscopy. Sci. Chin. Chem..

[15] Shang G., Li C., Wen G., Zhang X., Liang A., Jiang Z. (2016). A new silver nanochain SERS analytical platform to detect trace hexametaphosphate with a rhodamine S molecular probe. Luminescence.

[16] Zhao W., Qian D., Zhang S., Li S., Inganas O., Gao F., Hou J. (2016). Fullerene-free polymer solar cells with over 11% efficiency and excellent thermal stability. Adv. Mater..

[17] Cai Q., Hu Z., Zhang Q., Li B., Shen Z. (2017). Fullerene (C_60_)/CdS nanocomposite with enhanced photocatalytic activity and stability. Appl. Surf. Sci..

[18] Baena J.R., Gallego M., Valcarcel M. (2002). Fullerenes in the analytical sciences. Trends Anal. Chem..

[19] Lanzellotto C., Favero G., Antonelli M.L., Tortolini C., Cannistraro S., Coppari E., Mazzei F. (2014). Nanostructured enzymatic biosensor based on Fullerene and gold nanoparticles:Preparation, characterization and analytical applications. Biosens. Bioelectron..

[20] Lu X., Shan D., Yang J., Huang B., Zhou X. (2013). Determination of m-dinitrobenzene based on novel type of sensor using thiol-porphyrin mixed monolayer-tethered polyaniline with intercalating fullerenols. Talanta.

[21] Hang L., Wang Q., Gao F., Shi J., Gao F. (2014). A high-performance DNA biosensor using polyhydroxylated fullerenol as 3D matrix for probe immobilization. Electrochem. Commun..

[22] Wu H., Fan S., Jin X., Zhang H., Chen H., Dai Z., Zou X. (2014). Construction of a Zinc Porphyrin–Fullerene-Derivative Based Nonenzymatic Electrochemical Sensor for Sensitive Sensing of Hydrogen Peroxide and Nitrite. Anal. Chem..

[23] Li R., Zhen M., Guan M., Chen D., Zhang G., Ge J., Gong P., Wang C., Shu C. (2013). A novel glucose colorimetric sensor based on intrinsic peroxidase-like activity of C_60_-carboxy fullerenes. Biosens. Bioelectron..

[24] Bhim B.P., Ragini S., Anil K. (2017). Synthesis of fullerene (C_60_-monoadduct)-based water-compatible imprinted micelles for electrochemical determination of chlorambucil. Biosens. Bioelectron..

[25] Li Q., Shao S.J. (2017). Progress on optical probes for hydrogen sulfate anion sensing. Chin. J. Anal. Chem..

[26] Biesaga M., Schmidt N., Seubert A. (2004). Coupled ion chromatography for the determination of chloride, phosphate and sulphate in concentrated nitric acid. J. Chromatogr. A.

[27] Ali D.S., Faizullah A.T. (2012). Combination of FIA-CL technique with ion-exchanger for determination of sulphate in various water resources in Erbil city. Arab. J. Chem..

[28] Kumar S.D., Maiti B., Mathur P.K. (2001). Determination of iodate and sulphate in iodized common alt by ion chromatography with conductivity detection. Talanta.

[29] Meneses S.R., Maniasso N., Zagatto E.A. (2005). Spectrophotometric flow-injection determination of sulphate in soil solutions. Talanta.

[30] Chattaraj S., Dast A.K. (1992). Indirect atomic absorption spectrometric determination of sulfate in human blood serum. Analyst.

